# Proper Cyclin B3 Dosage Is Important for Precision of Metaphase-to-Anaphase Onset Timing in *Caenorhabditis elegans*

**DOI:** 10.1534/g3.112.002782

**Published:** 2012-08-01

**Authors:** Maja Tarailo-Graovac, Nansheng Chen

**Affiliations:** Department of Molecular Biology and Biochemistry, Simon Fraser University, Burnaby, British Columbia V5A 1S6, Canada

**Keywords:** *cyb-3* (Cyclin B3), dosage increase, MosSCI, anaphase onset variation, *mdf-1/MAD1*

## Abstract

Cyclin-dependent kinases (CDK) and their compulsory cofactors, the cyclins, are the two key classes of regulatory molecules that determine the eukaryotic cell's progress through the cell cycle by substrate phosphorylation. Cdk1 forms complexes with B-type cyclins and phosphorylates a number of substrates as cells prepare to enter mitosis. CYB-3 (Cyclin B3) is a B-type cyclin that has been recently shown to be required for the timely metaphase-to-anaphase transition, presumably by alleviating a spindle assembly checkpoint (SAC) block. Previously, we have shown that doubling the CYB-3 dosage suppresses sterility in the absence of the essential SAC component MDF-1/Mad1. Here we demonstrate the importance of the Mos1-mediated single-copy insertion method for understanding the effects of gene dosage by generating strains that have more (two or three) copies of the *cyb-3* in wild-type and *mdf-1(gk2)* backgrounds to investigate dosage effect of CYB-3 on mitotic progression as well as development and fertility in the absence and the presence of the MDF-1 checkpoint component. We show that tripling the dosage of CYB-3 results in a significantly variable metaphase-to-anaphase transition, both in wild-type and *mdf-1(gk2)* mutant backgrounds. Although a majority of embryos initiate anaphase onset normally, a significant number of embryos initiate anaphase with a delay. We also show that tripling the dosage of CYB-3 has no effect on viability in the wild-type background; however, it does reduce the sterility caused by the absence of MDF-1. Together, these data reveal that proper dosage of CYB-3 is important for precision of timely execution of anaphase onset regardless of the presence of the MDF-1 checkpoint component.

## Introduction

The progression through the stages of the eukaryotic cell cycle is temporally controlled by association of the cyclins to their corresponding cyclin-dependent kinases (CDK) (Sanchez and Dynlacht 2005; Satyanarayana and Kaldis 2009). Cyclins are expressed and most stable during the stages of the cell cycle when they are required. Thus, proper expression and degradation of cyclins are critical for controlled cell-cycle progression. Cyclin B levels, for example, rise in G2 phase of the cell cycle and decrease substantially at the metaphase-to-anaphase transition (Sanchez and Dynlacht 2005). In agreement with their expression profiles, activation of the CDK1 kinase by B-type cyclins triggers a cell to enter the M phase of the cell cycle, whereas inactivation of the CDK1 is required for exit from the M phase. In particular, B-type cyclins activate CDK1 to phosphorylate specific set of substrates, leading to proper chromosome condensation, centrosome maturation, and nuclear envelope breakdown (NEBD) as a cell prepares to partition the replicated genetic material to daughter cells (Blethrow *et al.* 2008).

Faithful segregation of chromosomes is ensured by the spindle assembly checkpoint (SAC), which monitors the status of kinetochore-microtubule attachment for proper chromosome attachment and tension state (May and Hardwick 2006; Musacchio and Salmon 2007). In the presence of improperly attached and tension-free chromosomes, the SAC is activated to delay anaphase onset by inhibiting the anaphase-promoting complex/cyclosome (APC/C), which thus stabilizes securin (May and Hardwick 2006; Musacchio and Salmon 2007). Once all the chromosomes have been properly attached to the spindle, the SAC needs to be silenced for timely anaphase onset to occur (Vanoosthuyse and Hardwick 2009). For instance, unattached kinetochores activate the SAC by recruiting the Mad2 component of the SAC to the kinetochores first (Waters *et al.* 1998; Essex *et al.* 2009). Once all of the kinetochores have achieved the proper attachment, the SAC is silenced by the minus-end–directed protein dynein, which “walks” away the Mad2 and other SAC components from kinetochores along mictotubules to centrosomes (Griffis *et al.* 2007; Howell *et al.* 2001; Schmidt *et al.* 2005; Sivaram *et al.* 2009). If the removal of the SAC components by dynein is compromised, the SAC remains activated even when the proper attachment is achieved, leading to unnecessary delay in anaphase onset due to the inhibition of APC/C activity.

In *Caenorhabditis elegans*, the SAC components (MAD1, MAD2, MAD3, BUB1, and BUB3) and the SAC regulation of the APC/C activity are conserved (Kitagawa and Rose 1999; Oegema *et al.* 2001; Nystul *et al.* 2003; Tarailo *et al.* 2007b; Kitagawa 2009a). Cyclin B is one of the key targets of the APC/C. In mammals, there are three B-type cyclins—B1, B2, and B3 (Gallant and Nigg 1994). Similarly, *C. elegans* has *cyb-1*, *cyb-2.1/2.2*, and *cyb-3* (van der Voet *et al.* 2009) B-type cyclins, which were shown to have both overlapping and distinct functions in chromosome segregation (van der Voet *et al.* 2009; Deyter *et al.* 2010). In both systems, cyclins B1 and B2 were shown to be highly similar, whereas cyclin B3 displayed more sequence conservation among the B3 proteins from other species than with the B1 and B2 proteins from the same species (Nguyen *et al.* 2002; Nieduszynski *et al.* 2002; van der Voet *et al.* 2009). In *C. elegans*, CYB-3 plays an essential role because the absence of CYB-3 by RNAi depletion (van der Voet *et al.* 2009; Deyter *et al.* 2010) or a gene knockout (Tarailo-Graovac *et al.* 2010) results in lethality. In particular, CYB-3 depletion leads to persistent block in the anaphase onset initiation (van der Voet *et al.* 2009; Deyter *et al.* 2010). Recently, it was shown that inability of *cyb-3(RNAi)* embryos to initiate anaphase onset is due to the compromised dynein-dependent removal of the SAC components from the kinetochores (Deyter *et al.* 2010).

Previously, the power of genetic screens was exploited to discover genetic interactors of *mdf-1/MAD1* and additional players in the SAC cascade by identifying suppressors (Tarailo *et al.* 2007a) and enhancers (Tarailo *et al.* 2007b) of the *mdf-1(gk2)* lethal phenotype. In *C. elegans*, the absence of MDF-1/Mad1–conserved SAC component leads to chromosome instability (CIN), accumulation of genetic errors, and ultimate death of *mdf-1(gk2)* worms in the F_3_ generation (Kitagawa and Rose 1999). So far, the majority of the mutants isolated from the suppressor screens proved to be lesions in the APC/C components that delayed anaphase onset and suppressed *mdf-1(gk2)* sterility (Kitagawa *et al.* 2002; Tarailo *et al.* 2007a). However, one of the cloned suppressors was shown to be due to doubling the CYB-3 dosage as a result of tandem duplication (Tarailo *et al.* 2007a; Zhao *et al.* 2008; Tarailo-Graovac *et al.* 2010). Interestingly, this was the first cloned suppressor of *mdf-1(gk2)* sterility that does not cause a constant delay in anaphase onset (Tarailo-Graovac *et al.* 2010). In this study, using the Mos1-mediated single-copy insertion method (MosSCI) (Frokjaer-Jensen *et al.* 2008), we created necessary strains and investigated dosage effect of CYB-3 on anaphase onset in strains that contain one, two, or three copies of the *cyb-3* in wild-type and the *mdf-1(gk2)* backgrounds. We show that proper CYB-3 dosage is essential for the precision of anaphase onset.

## Materials and Methods

### Strains and culturing conditions

The following mutant alleles were used in this work: *mdf-1(gk2)*, *unc-46(e177)*, *unc-119(ed3)*, *ttTi5605*, *dotSi100*, *cxTi10882*, *dotSi110*, *cyb-3(gk195)*, *such-4(h2168)*, *nT1[qIs51]*, *nT1[let-?(m435)]*, and *ruIs32*. The following strains were used in this work: N2 (Bristol strain as a wild-type), EG4322 [*unc-119(ed3) III*; *ttTi5605 II*]; EG6250 [*unc-119(ed3) III*; *cxTi10882 IV*], VC388 [*cyb-3(gk195) V/nT1[qIs51] (IV;V)*]; KR4233 [*unc-46(e177) mdf-1(gk2) such-4(h2168)*]; KR3627 [*unc-46(e177) mdf-1(gk2) V/nT1[let-?(m435)] (IV;V)*]. Additional strains used in this work were generated in this study using the standard genetic procedures. Strains were maintained using standard protocol on nematode growth media (NGM) plates seeded with OP50 bacteria (Brenner 1974). The strains were maintained at 20°, whereas the phenotypic analyses were performed at both 20° and 25° as noted in the Results section.

### Generating stable single-gene duplications using MosSCI

*cyb-3* locus was amplified using Phusion (NEB) high-fidelity DNA polymerase from the *C. elegans* N2 (Bristol) single worm lysates and cloned into the pCFJ178 vector (Frokjaer-Jensen *et al.* 2008). As described previously (Tarailo-Graovac *et al.* 2010), due to the toxic effect of the large amount of *cyb-3*, we co-injected 5 ng/μl of this targeting construct with the 50 ng/μL of Mos1 transposase pJL43.1 (*Pglh-2*::*transposase*), 5 ng/μL pGH8 (*Prab-3*::*mCherry*), 5 ng/μL pCFJ104 (*Pmyo-3*::*mCherry*), and 2.5 ng/μL (*Pmyo-2*::*mCherry*) into the gonad of 45 young adult P_0_ hermaphrodites of EG6250 [*unc-119(ed3) III*; *cxTi10882 IV*] strain. The plates that contained wild-type–looking *mCherry*–expressing worms were starved at 25° and then screened for stable integrants, as previously described (Frokjaer-Jensen *et al.* 2008). Once a stable line was obtained, it was confirmed by PCR and sequencing to contain a mutation-free wild-type copy of *cyb-3* inserted into the *cxTi10882* Mos1 site.

### Phenotypic analysis

For each analysis, hermaphrodites at L4 stage were grown on fresh OP50 plates at 20° or 25°. The hermaphrodites were transferred to fresh plates every 12 hr. Brood size was calculated based on the total eggs laid by each hermaphrodite. Embryos that did not hatch were scored as embryonic arrests, and the embryos that hatched but did not grow to adult stage were scored as larval arrests. The embryos that developed into adults were analyzed for the presence of males in all the strains analyzed, and the worms in the *mdf-1(gk2)* background were also analyzed for the percentage of the adult progeny that were sterile by individually plating all the adult progeny and observing for the presence or absence of offspring.

To assess whether the *dotSi110* could suppress the sterility of *mdf-1(gk2)* homozygotes, the F_1_ *mdf-1(gk2)* homozygotes segregated from KR3627 [*unc-46(e177) mdf-1(gk2) V/nT1[let-?(m435)] (IV;V)*] were mated to *dotSi110* males. The wild-type–looking *unc-46(e177) mdf-1(gk2)/ + +*; *dotSi110/ +* were allowed to self-fertilize, and 43 Unc-46 progeny were plated individually. All of the worms that could be propagated for more than three generations were genotyped and confirmed to be homozygous for *dotSi110* duplication of *cyb-3*, whereas none of the worms that could not be propagated for more than three generations were homozygous for the *dotSi110* duplication.

### Time-lapse imaging of the embryos

One-day old gravid adult hermaphrodites were dissected, and embryos were mounted onto 3% agarose pads as described (Sulston *et al.* 1983). Quorum WaveFX Spinning Disk system mounted on Zeiss Axioplan microscope was used. Early embryonic cell division was recorded using time-lapse video microscopy at 400× with 200 ms fluorescent exposure, one image every 10 sec. Image acquisition and analysis was performed using Volocity software.

## Results and Discussion

### Mos1-mediated single-copy insertion method to investigate increased dosage of CYB-3 in *Caenorhabditis elegans*

**Figure 1:**
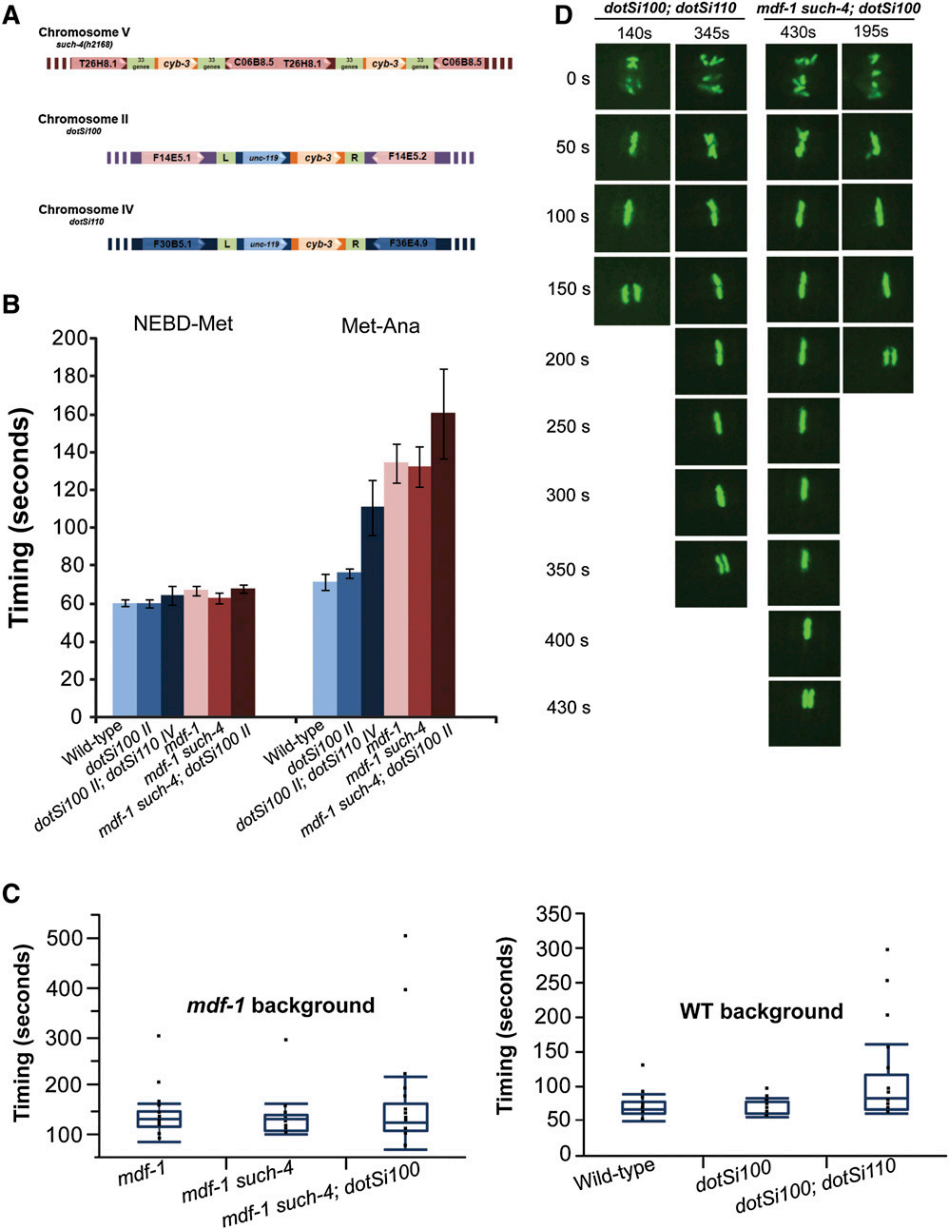
Increased variation in anaphase onset when dosage of CYB-3 is tripled using the MosSCI insertion method. (A) Schematic drawing of the *cyb-3* duplication locations in the *C. elegans* genome. *such-4(h2168)* is a tandem duplication that affects the region between the T26H8.1 and C06B8.5 nearly identical transposons. *dotSi100* is a *cyb-3* duplication that was inserted into the *ttTi5605* Mos1 insertion site on chromosome II between the F14E5.1 and the F14E5.2 genes. *dotSi110* is a *cyb-3* duplication that was inserted into the *cxTi10882* Mos1 integration site on chromosome IV between the F30B5.1 and the F36E4.9 genes. (B) Summary of the timing measurements from NEBD-to-metaphase and metaphase-to-anaphase intervals given in seconds. Error bars represent SEM for *n* = 20 measurements for each strain. (C) Boxplots of the metaphase-to-anaphase interval timing data for the embryos with one, two, or three copies of the *cyb-3* in the wild-type background (right) and the *mdf-1(gk2)* background (left). For each boxplot, all the data points (n = 20) were used. (D) *dotSi100*; *dotSi110*: An example of an embryo with normal timing of the anaphase onset (140 sec) and delayed anaphase onset (345 sec) in the wild-type background. *mdf-1 such-4*; *dotSi100*: An example of an embryo with normal timing of the anaphase onset (195 sec) and delayed anaphase onset (430 sec) in the *mdf-1(gk2)* background.

Our recent work has demonstrated that doubling the dosage of a *cyb-3* gene (Cyclin B3) suppressed the sterility in the absence of an essential SAC component MDF-1/Mad1 and allowed for the *mdf-1(gk2)* homozygotes to be propagated well beyond the third generation (Tarailo-Graovac *et al.* 2010). This finding clearly showed that in addition to the complete absence of the CYB-3 due to *cyb-3* gene knockout (Tarailo-Graovac *et al.* 2010) or RNAi depletion of the *cyb-3* gene product (van der Voet *et al.* 2009; Deyter *et al.* 2010), dosage of the functional CYB-3 may play an important role in the SAC cascade. Phenotypic analysis of a strain that is homozygous for the *cyb-3* duplication revealed that unlike other suppressors of the *∆mdf-1* sterility (Tarailo *et al.* 2007a), *cyb-3* duplication did not result in any obvious phenotypes in the wild-type background (Tarailo-Graovac *et al.* 2010). However, the titrations of multiple different concentrations at which the *cyb-3* constructs were injected into the gonads led us to conclude that high copy number of the *cyb-3*–containing constructs resulted in sterility, suggesting a dominant-negative effect of *cyb-3* overexpression (Tarailo-Graovac *et al.* 2010). To further investigate the effect of increased dosage of CYB-3 in *C. elegans* and to reach better understanding of the mode of genetic interaction between the *cyb-3* and *mdf-1*, we used the MosSCI method (Frokjaer-Jensen *et al.* 2008) to integrate a wild-type copy of the *cyb-3* gene at multiple sites in the *C. elegans* genome. Previously, we created a strain that contains a wild-type copy of the *cyb-3* gene integrated on chromosome II, in the *ttTi5605* Mos1 integration site (Tarailo-Graovac *et al.* 2010). To assess the effect of three copies of the *cyb-3* gene, we first inserted a copy of the *cyb-3* gene on chromosome IV, in the *cxTi10882* Mos1 integration site, and created the *dotSi110* stable insertion in addition to the previously generated *dotSi100* stable insertion on chromosome II (Tarailo-Graovac *et al.* 2010) (Figure 1A). Once confirmed by sequencing that the wild-type mutation-free copy of *cyb-3* gene was inserted on chromosome IV, we assessed the functionality of *cyb-3* from the *dotSi110* by testing its ability to rescue the *gk195* lethality. As we have shown previously, the *gk195* knockout allele removes the majority of *cyb-3* and results in larval arrest (Tarailo-Graovac *et al.* 2010). As with the stable integration of *cyb-3* on chromosome II, we were able to construct the *cyb-3(gk195) V*; *cyb-3(dotSi110) IV* homozygotes and to show that *dotSi110* fully rescues the larval arrest phenotype of the *cyb-3(gk195)* knockout allele, making *cyb-3(gk195) V*; *cyb-3(dotSi110) IV* homozygotes indistinguishable from the wild-type N2 strain at 20° and 25° (data not shown). This result confirms that the wild-type copy of *cyb-3* integrated on chromosome IV is expressed and makes fully functional CYB-3. Next, we compared the development of *dotSi110* duplication-containing worms to the *dotSi100* duplication-containing worms for developmental defects, such as developmental delay, increased incidence of embryonic or larval arrests, sterility and morphological defects at 20° and 25° (Table 1). Our analysis revealed no obvious difference between the two duplications (Table 1). Furthermore, this analysis confirmed our previous conclusions that, although overexpression of *cyb-3* in high-copy extrachromosomal arrays is toxic, a duplication of *cyb-3* does not have an obvious phenotype in an otherwise wild-type background in *C. elegans*. Finally, we compared the ability of the *dotSi110* duplication to suppress lethality of the *mdf-1(gk2)* homozygotes. We observed that all of the 21 analyzed *∆mdf-1*; *dotSi110* homozygotes could be propagated indefinitely. Then we analyzed whether the *dotSi110* duplication behaves the same in the *mdf-1(gk2)* background as the previously characterized *such-4(h2168)* tandem duplication (Tarailo *et al.* 2007a; Zhao *et al.* 2008; Tarailo-Graovac *et al.* 2010) and the *dotSi100* duplication generated on chromosome II (Tarailo-Graovac *et al.* 2010). We observed that *dotSi110* duplication has a very similar phenotype to the *such-4(h2168)* (Table 2) and *dotSi100* duplications (Tarailo-Graovac *et al.* 2010). Together, these data suggest that increased dosage of CYB-3 has similar effect regardless of analyzed chromosomal positions of the duplicated *cyb-3* gene, which makes the MosSCI a valuable tool for gene dosage studies (Figure 1A).

**Table 1:** *cyb-3* dosage effect in the wild-type background

Genotype	*cyb-3* Copies	T	Embryonic Arrest (%)	Larval Arrest (%)	Adult (%)	Male (%)
*dotSi101 II [unc-119( +)]* (*n* = 859)	One	20°	7.0	4.1	88.9	0.1
*dotSi100 II [T06E6.2 + unc-119( +)]* (*n* = 4116)	Two	20°	1.8	0.8	97.4	0.0
*dotSi110 IV [T06E6.2 + unc-119( +)]* (*n* = 3741)	Two	20°	1.4	0.7	97.9	0.2
*dotSi100 II*; *dotSi100 IV* (*n* = 4599)	Three	20°	1.1	0.5	98.4	0.1
*dotSi101 II [unc-119( +)]* (*n* = 859)	One	25°	7.8	1.3	90.9	0.0
*dotSi100 II [T06E6.2 + unc-119( +)]* (*n* = 4116)	Two	25°	4.8	2.4	92.8	0.3
*dotSi110 IV [T06E6.2 + unc-119( +)]* (*n* = 3741)	Two	25°	4.6	3.3	92.1	0.5
*dotSi100 II*; *dotSi100 IV* (*n* = 4599)	Three	25°	2.0	3.5	94.5	0.5

**Table 2:** *cyb-3* dosage effect on the *mdf-1(gk2)* lethality

Genotype	*cyb-3* Copies	Embryonic Arrest (%)	Larval Arrest (%)	Adult (%)	Fertile (%)	Male (%)
*unc-46(e177) mdf-1(gk2) such-4(h2168)* (*n* = 927)	Two	43.8	27.9	28.3	52.8	1.5
*unc-46(e177) mdf-1(gk2)*; *dotSi110* (*n* = 1010)	Two	52.5	26.3	21.2	54.5	2.1
*unc-46(e177) mdf-1(gk2) such-4(h2168)*; *dotSi100* (*n* = 2460)	Three	43.0	26.5	30.5	77.3	5.5
*unc-46(e177) mdf-1(gk2)*; *dotSi100*; *dotSi110* (*n* = 726)	Three	41.4	26.3	32.3	71.9	4.7

### An effect of further increase in the *cyb-3* gene number on development in the presence and the absence of the functional MDF-1/Mad1 checkpoint component

The MosSCI (Frokjaer-Jensen *et al.* 2008) method allows us to construct the strains with the desired number of copies of the gene of interest and follow the consequences of increased dosage on development of the whole organism in different genetic backgrounds. Once we confirmed the functionality of both of the MosSCI-engineered duplications *in vivo*, we constructed a strain that has three copies of the *cyb-3* gene: an endogenous *cyb-3* located on chromosome V, *dotSi100* integrated copy of *cyb-3* located on chromosome II, and the *cyb-3* integrated on chromosome IV (*dotSi110 IV*) (Figure 1A). To assess the effect of three copies of the *cyb-3* gene, we first investigated the development of these worms at 20° and 25° (Table1). Our analysis revealed that the animals that have three copies of the *cyb-3* gene develop normally at these temperatures, as they do not display any obvious difference from the wild-type or duplication strains (Table 1). Thus, our results suggest that there may be a threshold to the amount of CYB-3 dosage increase at which animals that bear too many *cyb-3* gene copies become sterile and uncoordinated, the phenotype observed when concentration of the *cyb-3*–containing constructs is greater than 5 ng/μL. However, a 3-fold increase in the CYB-3 dosage, like the 2-fold increase (Tarailo-Graovac *et al.* 2010), is well tolerated throughout the development in the wild-type background.

The *such-4* suppressor of the *mdf-1(gk2)* sterility is the weakest suppressor isolated to date (Tarailo *et al.* 2007a). Similar to the *cyb-3*–containing tandem duplication (Figure 1A), the MosSCI-engineered *cyb-3* duplications are very weak suppressors of *mdf-1(gk2)* sterility (Table 2). To investigate whether three copies of the *cyb-3* display any difference in suppressing the *mdf-1(gk2)* lethal phenotype, we introduced the *dotSi100* and *dotSi110* single-copy *cyb-3* stable integrations into the *mdf-1(gk2)* background using standard genetic crosses followed by genotyping procedures. Phenotypic analysis of the *unc-46(e177) mdf-1(gk2)*; *dotSi100*; *dotSi110* strain revealed 41.1% of embryonic arrests, 26.3% of larval arrests, and 32.3% of progeny that developed into adults (Table 2). These results are very similar to the percentages of developmental arrests and adult progeny that we observed when analyzing either a tandem duplication *such-4(h2168)* or a *cyb-3* duplication located on either chromosome IV (Table 2) or II (Tarailo-Graovac *et al.* 2010), suggesting that tripling the CYB-3 dosage does not affect *mdf-1(gk2)* lethality. However, we did observe a difference in percentage of adult progeny that were fertile. Whereas in the strains that contained duplicated *cyb-3* in the *mdf-1(gk2)* background, some 50% of fertile adult progeny was fertile, in the *unc-46(e177) mdf-1(gk2)*; *dotSi100*; *dotSi110* strain, the number of fertile progeny was 71.9% (Table 2). To ensure that the observed increase in the fertile adult progeny was due to an extra *cyb-3* copy, we constructed two additional strains: *unc-46(e177) mdf-1(gk2) such-4(h2168)*; *dotSi100* and *unc-46(e177) mdf-1(gk2) such-4(h2168)*; *dotSi110*. In both strains, the percentage of fertile progeny was over 70% (Table 2 and data not shown), which is greater than in the strains that contained *cyb-3* duplications in the *mdf-1(gk2)* background (Table 2; Tarailo-Graovac *et al.* 2010). Thus, these data suggest that increasing the *cyb-3* copy number from two to three suppresses the sterility of the *mdf-1(gk2)* homozygotes further by increasing the percentage of adult progeny that are fertile.

### An effect of three copies of *cyb-3* gene on metaphase-to-anaphase transition in the presence and the absence of the functional MDF-1/Mad1 checkpoint component

To date, the *such-4* suppressor is the only cloned suppressor that allows indefinite propagation of the *mdf-1(gk2)* homozygotes without having any obvious anaphase onset delays (Tarailo-Graovac *et al.* 2010). An additional three suppressors whose molecular identity is currently unknown belong to this class (Tarailo *et al.* 2007a). To further investigate the effect of the CYB-3 dosage on anaphase onset timing, we decided to analyze the cell-cycle progression in the strains that contain three copies of the *cyb-3* gene. We reasoned that further increase in the CYB-3 dosage might result in more obvious alteration of anaphase onset. Similar to the analysis of the duplication strains, we introduced all of the analyzed strains into a *ruIs32* background (Table 3), which is an integrated histone-GFP transgene that marks mitotic chromosome behavior (Praitis *et al.* 2001). Next, we used time-lapsed fluorescence microscopy of one-cell–stage embryos to measure the time it takes an embryo to progress from complete nuclear envelope breakdown (NEBD) to anaphase onset (Table 3). As expected, we did not observe any significant difference in the anaphase onset timing between wild-type (131.6 sec, *n* = 20) and *dotSi110cyb-3* duplication-containing embryos (135.0 sed, *n* = 10) (Table 3). We also observed the similar NEBD-to-anaphase onset progression in the control embryos (*dotSi101*), which have the *unc-119* rescue construct integrated without the wild-type copy of the *cyb-3* gene and the *dotSi100cyb-3* duplication embryos (Table 3). These results agree well with our previously published analysis (Tarailo-Graovac *et al.* 2010). Unlike the timing in the strains that contain two copies of the *cyb-3* gene, the strain that had three copies had a very variable anaphase onset (Figure 1, C and D; Table 3). In these embryos, the anaphase onset timing ranged from 120 to 450 sec, which is very different from the wild-type strain, in which the range was very small (110 to 190 sec). Although the majority of the embryos that contained three copies of the *cyb-3* had normal anaphase onset timing, the 25% of embryos that divided with the delay (Figure 1, C and D) made an overall difference in the NEBD-to-anaphase onset progression significant (175.2 sec, *n* = 20; *P* = 0.0291) (Table 3).

**Table 3:** *cyb-3* dosage effect on anaphase onset in wild-type and *mdf-1(gk2)* backgrounds

Genotype	*cyb-3* Copies	NEBD-to-Anaphase Onset sec ± SEM (n)
Wild-type background		
* unc-119(ed3)*; *ruIs32(pie-1*::*H2B-GFP)*	One	131.6 ± 4.3 (20)
* dotSi101 II [unc-119( +)]*; *ruIs32*	One	136.8 ± 4.6 (10)
* dotSi100 II [T06E6.2 + unc-119( +)]*; *ruIs32*	Two	135.6 ± 2.7 (20)
* dotSi110 IV [T06E6.2 + unc-119( +)]*; *ruIs32*	Two	135.0 ± 3.0 (10)
* dotSi100*; *dotSi110*; *ruIs32*	Three	175.2 ± 18.7*^a^* (20)
*mdf-1* background		
* *F_2_ *unc-46(e177) mdf-1(gk2)*; *ruIs32*	One	201.3 ± 10.7*^a^* (20)
* unc-46(e177) mdf-1(gk2) such-4(h2168)*; *ruIs32*	Two	195.2 ± 11.3*^a^* (21)
* unc-46(e177) mdf-1(gk2) such-4(h2168)*; *dotSi100*; *ruIs32*	Three	226.5 ± 22.9*^a^* (20)

a Significant difference from the measurements in the *unc-119(ed3)*; *ruIs32(pie-1*::*H2B-GFP)* background was determined using the unpaired Student *t*-test.

Next, we asked whether the same variability in the anaphase onset timing is also observed when there are three copies of the *cyb-3* gene in the *mdf-1(gk2)* background (Table 3). In agreement with our previous results, we observed a constant, significant delay in the embryos that lacked MDF-1 compared with the wild-type embryos (Tarailo-Graovac *et al.* 2010) (Table 3). Whereas the *cyb-3* duplication strains analyzed in the *mdf-1(gk2)* background did not progress through anaphase onset with any additional delays, when compared with the *mdf-1(gk2)* embryos alone, the strain that contained three copies of the *cyb-3* displayed a highly variable progression through anaphase onset (Figure 1, C and D; Table 3). Similar to the embryos analyzed in the wild-type background, the anaphase onset timing of the embryos that had three copies of the *cyb-3* in the *mdf-1(gk2)* background displayed a large range (144 to 570 sec) (Figure 1, C and D; Table 3), which is very different from the range observed in the *mdf-1(gk2)* homozygotes (150 to 368 sec) (Figure 1, C and D; Table 3). Similar to the analysis in the wild-type background, the majority of the embryos (75%) that contained three copies of the *cyb-3* in the *mdf-1(gk2)* background progressed to anaphase with the timing similar to the *mdf-1(gk2)* homozygotes; however, 25% of embryos divided with the significant delay. To confirm that the highly variable anaphase onset is due to three copies of the *cyb-3* gene, we constructed the *unc-46(e177) mdf-1(gk2)*; *dotSi100*; *dotSi110*; *ruIs32* and *unc-46(e177) mdf-1(gk2) such-4(h2168)*; *dotSi110*; *ruIs32* strains. In both of these strains, we also observed the similarly variable anaphase onset as that observed in the *unc-46(e177) mdf-1(gk2) such-4(h2168)*; *dotSi100*; *ruIs32* strain (data not shown). In summary, these results show that increasing the dosage of CYB-3 3-fold results in highly variable NEBD-to-anaphase onset progression that is independent of the presence or the absence of the SAC component MDF-1.

The observed variability in NEBD-to-anaphase onset progression may be due to a delay in alignment of chromosomes on the metaphase plate or due to a delay in initiation of anaphase onset. To distinguish between the two, we measured the intervals from NEBD-to-metaphase plate formation and from metaphase-to-anaphase transition (Figure 1B). In all the embryos analyzed in either the wild-type or the *mdf-1(gk2)* backgrounds, we observed little variation in the formation of the metaphase plates (Figure 1B). Unlike the time it takes for the chromosomes to align at the metaphase plate, the metaphase-to-anaphase interval in the strains that had three copies of the *cyb-3* gene was very variable, regardless of the presence or the absence of the MDF-1 checkpoint component (Figure 1, B and D). To assess a significance of the observed variation in metaphase-to-anaphase transition, we compared the boxplots of the timing results between different strains (Figure 1C). These boxplots clearly show the tendency of time for the anaphase onset in the strains that contain triple the amount of CYB-3, regardless of the presence of the MDF-1 checkpoint component, to be skewed toward the longer anaphase onset (Figure 1C). To further test whether the observed variation in metaphase-to-anaphase transition was significant, we used Bartlett's test to probe the variances from the strains analyzed. We determined that the variances in anaphase onset timing between a duplication strain and the wild-type strain were not significantly different (*P* = 0.0572). We also calculated that variances in anaphase onset timing between a duplication strain in the *mdf-1(gk2)* background and the *mdf-1(gk2)* homozygotes were not significantly different (*P* = 0.575). Importantly, the anaphase onset timing of the strains that contained three copies of the *cyb-3* in either the wild-type (*P* = 8.46E−07) or *mdf-1(gk2)* background (*P* = 7.84E−04) was significantly more variable than in the wild-type or *mdf-1(gk2)* homozygous strain alone. Therefore, our data show that increasing the copy number of *cyb-3* to three resulted in significantly variable anaphase onset that was independent of the presence of the functional MDF-1 SAC component. Whereas in the wild-type background the observed variation in anaphase onset does not affect viability of the strain, in the *mdf-1(gk2)* background, the delay in anaphase onset in 25% of the embryos might actually be the reason for the observed increase in fertility.

In contrast to the mammalian SAC components, absence of a functional SAC component in *C. elegans* due to either RNAi depletion or gene knockout does not cause precocious anaphase onset (Encalada *et al.* 2005; Tarailo *et al.* 2007a; Tarailo-Graovac *et al.* 2010). In contrast, absence of BUB-1 and MDF-1 was shown to extend the metaphase-to-anaphase transition (Encalada *et al.* 2005; Tarailo-Graovac *et al.* 2010). Furthermore, albeit the SAC components were shown to be required to delay anaphase onset in the presence of microtubule depolymerizing drug nocodazole (Encalada *et al.* 2005), the SAC-dependent anaphase onset delay is very small compared with the delays experienced by the mammalian cells in the presence of compromised attachment of kinetochores to the spindle (Waters *et al.* 1998). Whereas the anaphase onset may be delayed up to several hours in mammals (Waters *et al.* 1998), the delays observed using the early *C. elegans* embryonic cells were relatively small, 2- to 3-fold, even in the presence of the severe spindle damage after nocodazole treatment (Kitagawa and Rose 1999; Encalada *et al.* 2005; Essex *et al.* 2009; Kitagawa 2009b). One of the reasons for such a difference may be the absolute requirement of the asynchrony of cell division for proper development and cell fate specification. It is likely that in *C. elegans* longer than 2- to 3-fold delays in M phase would interfere with asynchrony of cell division and would ultimately lead to lethality. Thus, one would expect the cell to balance between the time required for a cell experiencing aberrant attachment of chromosomes to the spindle to correct the defects and the time that cannot be exceeded for the asynchrony of the cell division to be maintained.

Recently, it was shown that in CYB-3–depleted embryos the SAC-dependent anaphase onset delay is extended well beyond the previously observed 2- to 3-fold delays due to the inability of the CYB-3–depleted embryos to silence the activated SAC signal (Deyter *et al.* 2010). In this article, we document an exciting discovery that tripling the CYB-3 dosage resulted in significant variation in anaphase onset skewed toward the delay in anaphase onset. In the 25% of embryos that divided with delayed anaphase onset, the delay did not exceed the previously reported 2- to 3-fold increase (Figure 1D), which may explain the lack of lethality in the wild-type background (Table 1). On the other hand, the observed delays may explain the increase in the percentage of fertile progeny in the absence of the MDF-1 checkpoint component when the dosage of CYB-3 is increased from 2-fold to 3-fold (Table 2). Furthermore, an interesting discovery is that the observed delays in the 25% of embryos were not dependent on the presence of the functional SAC component MDF-1, which may suggest that the observed delays might be caused by activation of another SAC component. In agreement with this hypothesis, Schumacher and colleagues reported that in the absence of MDF-1, the prolonged metaphase in the *cyb-3 (RNAi)* embryos was abolished, resulting in anaphase onset; however, the anaphase onset was nonetheless delayed (Deyter *et al.* 2010).

## Conclusion

The MosSCI represents an invaluable tool for studying the consequence of altered gene dosage on development of a multicellular organism in different genetic backgrounds. Using this method, we were able to create necessary strains and compare the effects of one, two, and three copies of the *cyb-3* gene on timely progression through mitosis and development in the wild-type and *mdf-1(gk2)* backgrounds. Our analysis revealed significant variation in the anaphase onset in the strains that had three copies of the *cyb-3*. The observed variation was independent of the functional MDF-1 SAC component and was skewed toward delayed anaphase onset. The increased CYB-3 dosage did not seem to affect proper development in the wild-type background, presumably because, even though significant, the observed delays did not extend the 2- to-3-fold increase in timing, which would not interfere with asynchrony of cell division and proper development. However, in the *mdf-1(gk2)* background, the increased CYB-3 dosage results in increased fitness.
